# THE PERSISTENCE OF TRADITIONAL VALUES: GRANDPARENTS REARING GRANDCHILDREN

**DOI:** 10.1093/geroni/igz038.2479

**Published:** 2019-11-08

**Authors:** Anne B Edwards, Tammy L Henderson, Crystal Niemeyer, Jessica L Stanley

**Affiliations:** 1 Purdue University Northwest, Hammond, Indiana, United States; 2 Lamar University, Beaumont, Texas, United States; 3 University of Oklahoma, Tulsa, OK, United States

## Abstract

The goal of this study is to examine how cultural values are preserved and transmitted by grandparents rearing grandchildren in one community in the southeast region of the Yukon-Koyukuk Census Area in Alaska. The eight participants (six females and two males) lived in a community in the Kusilvak Census Area, with ages ranging from 47 to 73 years old. Participants’ took part in a semi-structured interview, which were then transcribed and coded into larger themes of 1) loss of traditional values, 2) continuing traditional values, 3) practicing traditional values, and 4) transmitting traditional values. The participants provided examples of how the cultural values that were strong at one point in their lives, were no longer exemplified in their community, and, in fact, behaviors that went against accepted values were seen. Participants spoke most often of how community members were cared for, how the community was valued over the individual, and the connections within families. The GRGs practiced those traditional values by caring, supporting, and loving the people in their families and communities, and by practicing humor and sharing with others. While this community has been influenced by modern ways of living currently found in the United States and Canada, it still remains relatively isolated from the technological and social influences that dominate what is considered “typical, modern” family life. The findings from this study illustrate the important roles that GRGs play in the persistence of cultural values, and the importance of incorporating these values in programs to assist this community.

